# Development of Artificial Intelligence‐Assisted Lumbar and Femoral BMD Estimation System Using Anteroposterior Lumbar X‐Ray Images

**DOI:** 10.1002/jor.70000

**Published:** 2025-07-09

**Authors:** Toru Moro, Noriko Yoshimura, Taku Saito, Hiroyuki Oka, Sigeyuki Muraki, Toshiko Iidaka, Takeyuki Tanaka, Kumiko Ono, Hisatoshi Ishikura, Naoya Wada, Kenichi Watanabe, Masayuki Kyomoto, Sakae Tanaka

**Affiliations:** ^1^ Division of Science for Joint Reconstruction, Graduate School of Medicine The University of Tokyo Tokyo Japan; ^2^ Sensory and Motor System Medicine, Faculty of Medicine Surgical Sciences, Graduate School of Medicine The University of Tokyo Tokyo Japan; ^3^ Department of Prevention Medicine for Locomotive Organ Disorders, 22nd Century Medical and Research Center The University of Tokyo Hospital Tokyo Japan; ^4^ Osteoporosis Center The University of Tokyo Hospital Tokyo Japan; ^5^ Division of Musculoskeletal AI System Development, Graduate School of Medicine The University of Tokyo Tokyo Japan; ^6^ Division of Therapeutic Development for Intractable Bone Diseases, Graduate School of Medicine and Faculty of Medicine The University of Tokyo Tokyo Japan; ^7^ Advanced Technology Research Institute Corporate R&D Group, KYOCERA Corporation Kawasaki Kanagawa Japan; ^8^ Medical R&D Center Corporate R&D Group, KYOCERA Corporation Yasu Shiga Japan

**Keywords:** artificial intelligence, bone density, bone mineral density, deep learning, osteopenia, osteoporosis

## Abstract

The early detection and treatment of osteoporosis and prevention of fragility fractures are urgent societal issues. We developed an artificial intelligence‐assisted diagnostic system that estimated not only lumbar bone mineral density but also femoral bone mineral density from anteroposterior lumbar X‐ray images. We evaluated the performance of lumbar and femoral bone mineral density estimations and the osteoporosis classification accuracy of an artificial intelligence‐assisted diagnostic system using lumbar X‐ray images from a population‐based cohort. The artificial neural network consisted of a deep neural network for estimating lumbar and femoral bone mineral density values and classifying lumbar X‐ray images into osteoporosis categories. The deep neural network was built by training dual‐energy X‐ray absorptiometry‐derived lumbar and femoral bone mineral density values as the ground truth of the training data and preprocessed X‐ray images. Five‐fold cross‐validation was performed to evaluate the accuracy of the estimated BMD. A total of 1454 X‐ray images from 1454 participants were analyzed using the artificial neural network. For the bone mineral density estimation performance, the mean absolute errors were 0.076 g/cm^2^ for the lumbar and 0.071 g/cm^2^ for the femur between dual‐energy X‐ray absorptiometry‐derived and artificial intelligence‐estimated bone mineral density values. The classification performances for the lumbar and femur of patients with osteopenia, in terms of sensitivity, were 86.4% and 80.4%, respectively, and the respective specificities were 84.1% and 76.3%.

**Clinical Significance:**

The system was able to estimate the bone mineral density and classify the osteoporosis category of not only patients in clinics or hospitals but also of general inhabitants.

## Graphical abstract

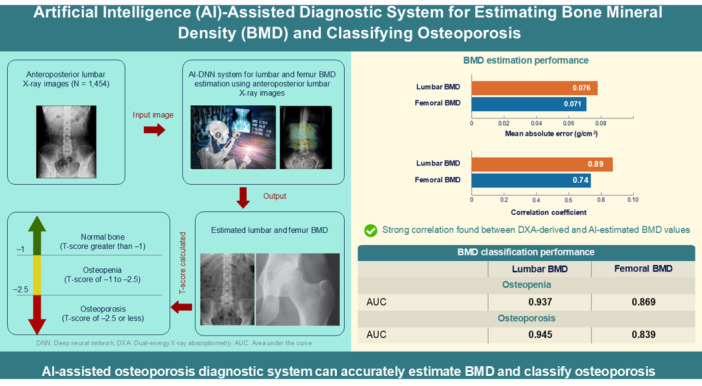

## 1 Introduction

Osteoporosis is a significant public health concern in the aging population that has tremendous social and economic costs worldwide. In 2000, there were 9.0 million osteoporotic fractures worldwide, including 1.4 and 1.6 million clinical vertebral and hip fractures, respectively, and the number of hip fractures was projected to increase to 2.6 million by 2020 and 4.5 million by 2050 [1]. The lifetime risks of any fracture of the hip, spine, or forearm in the United States (US) were estimated to be 40% in females and 13% in males [2]. The global economic cost of osteoporosis in 1998 was 34.8 billion US dollars, which is expected to increase to 131.5 billion US dollars by 2050 [3]. Such fragility fractures increase the risk of secondary fractures at other sites in addition to those at the same site [4]. Cumulative fractures in older adults are not only associated with reduced activities of daily living and quality of life [5] but also have a considerable impact on life expectancy [6]. Therefore, early diagnosis and treatment of osteoporosis and prevention of fragility fractures are urgent issues not only for healthcare but also for society.

Based on this social background, progress has been made in the prevention, diagnosis, and treatment of osteoporosis, including the establishment of consensus statements [7], Japanese guidelines [8], and successive clinical application of drugs [9]. However, in real‐world clinical practice, it is common to visit a hospital after a fragility fracture, receive a diagnosis of osteoporosis, and start treatment because osteoporosis is usually asymptomatic, and it is difficult to motivate patients to visit the clinic or hospital. Moreover, the medical care infrastructure for early diagnosis of osteoporosis needs to be improved. Although bone mineral density (BMD) measurements are recommended for the lumbar spine and proximal femur using dual‐energy X‐ray absorptiometry (DXA) for the diagnosis of osteoporosis [2, 8], DXA equipment is not commonly used [10]. However, radiographic equipment has a higher coverage rate than DXA equipment [11]. Additionally, approximately 619 million patients [12] undergo X‐ray examinations during the first orthopedic service. Therefore, patients with potential osteoporosis may be treated early, owing to an estimate of BMD from radiographic images of patients with any disease.

Several image diagnostic systems using artificial intelligence (AI) have recently been studied for various diseases [13, 14, 15, 16]. Images from lumbar computed tomography or dental panoramic radiography have been used to diagnose osteoporosis [17, 18]. Furthermore, several research groups have focused on plain X‐ray images to increase opportunities to identify patients with osteoporosis with more common radiographic findings [19, 20, 21, 22, 23, 24, 25]. We developed an AI‐assisted osteoporosis diagnostic system that outputs the estimated lumbar BMD from anteroposterior lumbar [26] and chest X‐ray images [27].

Here, we developed an AI‐assisted diagnostic system that outputs not only lumbar BMD but also femoral BMD from anteroposterior lumbar X‐ray images. We evaluated the performance of the lumbar and femoral BMD estimations and osteoporosis classification accuracy of the AI‐assisted diagnostic system using lumbar X‐ray images from a population‐based cohort. The hypotheses of this study are as follows: first, AI can estimate the BMD of not only the lumbar spine but also the femur from one lumbar plain anteroposterior X‐ray image and classify patients with osteopenia and osteoporosis. Second, the accuracy of femoral BMD estimation (not appearing in the X‐ray image) would be sufficient but not greater than that of lumbar BMD estimation (appearing in the X‐ray image). Third, AI can estimate the BMD of the general population and classify patients with osteopenia and osteoporosis.

## 2 Methods

### 2.1 Participants

**Figure 1:**
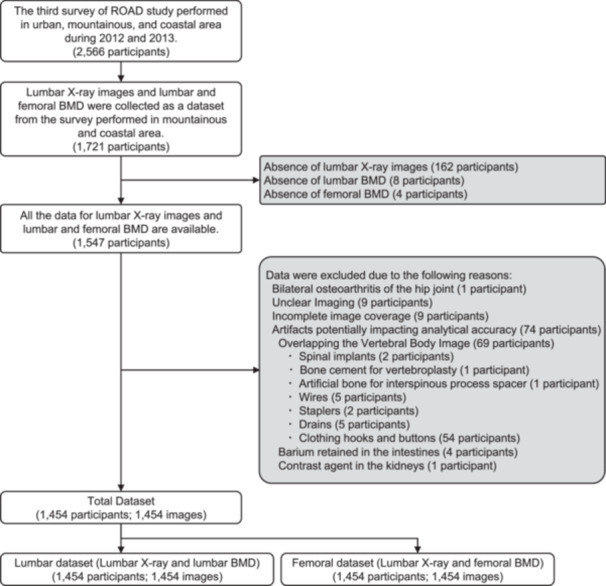
Study flowchart of lumbar X‐ray images included in the training and test data sets.

The “Research on Osteoarthritis/Osteoporosis Against Disability (ROAD)” study, which began in 2005, is a large‐scale population‐based prospective cohort study of osteoarthritis and osteoporosis in several communities in urban (Itabashi, Tokyo, Japan), mountainous (Hidakagawa, Wakayama, Japan), and coastal (Taiji, Wakayama, Japan) areas [28]. This study (level of evidence: 3) used the third survey data (2012–2013 [29]) of mountainous and coastal areas from the ROAD study. Invitation letters for the third survey were distributed to inhabitants whose names were listed in the two former surveys of the ROAD study. In addition to former participants, inhabitants aged ≥ 40 year who were willing to participate in the ROAD study were included. A study flowchart of lumbar X‐ray images is shown in Figure 1. A total of 1721 individuals (769 from mountainous areas and 952 from coastal areas) participated in the third visit. The exclusion criteria were cases of bilateral osteoarthritis of the hip joint (*n* = 1), as the BMD of the proximal femur may be measured higher than its actual value; cases of unclear imaging (*n* = 9) in which the entire image was unclear and the spine was difficult to identify with the naked eye; cases of incomplete image coverage (*n* = 9), where the imaging coverage did not include the T11–L5 vertebrae; and cases in which artifacts potentially affected the analytical accuracy (*n* = 69) (Among these, wires and staplers were excluded if their total length exceeded the transverse diameter of the L1 vertebral body. However, compression fractures, osteosclerosis, degeneration, and scoliosis of the spine were included. All the participants provided written informed consent, and this study was approved by the ethics committees of the University of Tokyo Hospital (11953).

### 2.2 X‐Ray Imaging and BMD Measurement

Plain radiographs in Digital Imaging and Communications in Medicine format of the lumbar spine in the anteroposterior view were obtained using two types of equipment manufactured by FUJIFILM Corporation (Tokyo, Japan) and KONICA MINOLTA Inc. (Tokyo, Japan). The BMD values were measured at the lumbar spine (L2–L4) and the proximal femur using a DXA device (Hologic Discovery; Hologic, Waltham, MA). The same DXA equipment was used for all participants. Osteopenia and osteoporosis were defined according to the World Health Organization criteria [30]: normal (T‐score ≥ −1.0), osteopenia (−2.5 < T‐score < −1.0), and osteoporosis (T‐score ≤ −2.5). The cutoff values were based on the Japanese guidelines for lumbar and femoral BMD values [8]. Osteopenia was defined as a BMD between 0.7135 and 0.8920 g/cm^2^ at the lumbar spine and between 0.5650 and 0.7000 g/cm^2^ at the femoral neck, regardless of sex. Osteoporosis was also defined by BMD of 0.7135 g/cm^2^ or lower at the lumbar spine and 0.5650 g/cm^2^ or lower at the femoral neck, regardless of sex.

### 2.3 Segmentation Network for Lumbar BMD Estimation

**Figure 2:**
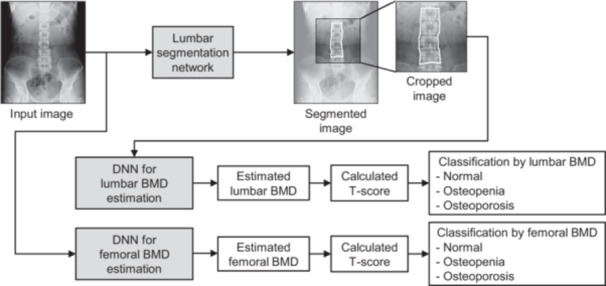
Illustration of the osteoporosis classification flowchart. BMD, bone mineral density; DNN, deep neural network.

The artificial neural network (ANN) for lumbar BMD estimation consisted of two stages: the first stage was the segmentation network for detecting the spinal area and the second stage was the deep neural network (DNN) for estimating the BMD value of the lumbar spine from the X‐ray image (Figure 2).

Image preprocessing for the segmentation network, which included cropping, rotation, and horizontal flipping, was randomly applied to the X‐ray images of the training data set to prevent network overfitting. The segmentation network was based on an encoder–decoder architecture to reduce computational cost [31]. The segmentation model was trained using preprocessed X‐ray images and annotation data, where the area of the L1–L4 lumbar vertebrae was marked as ground truth. Adaptive moment estimation was used as the optimization algorithm [32]. For femoral BMD estimation, only the DNN was used to estimate the BMD value of the femoral neck. A segmentation network was not implemented for this task, as the femoral neck did not appear on lumbar spine X‐ray images. Instead, BMD estimation for the femoral neck was performed using the entire image without prior segmentation.

### 2.4 Image Preprocessing and Data Augmentation

To improve the lumbar and femoral BMD estimation accuracy of the DNN, image preprocessing was performed before DNN training or BMD estimation. Data augmentation was conducted to increase the number of X‐ray images and obtain sufficient images to train the DNN. Data augmentation simulated imaging variations that could occur during actual radiography. Data augmentation was performed randomly for each X‐ray image, random type of change, and random number of images.

### 2.5 DNM

The ANN used consisted of the DNN to estimate the BMD of the lumbar or femur from an anteroposterior lumbar X‐ray image. A transformer‐based network was selected as the suitable network [33]. The mean squared error was adopted as the loss function to calculate the difference between the teacher data and the estimated BMD value during training. One layer in front of the loss‐function calculation unit was provided with a fully connected layer, and one scalar value was output as the estimated BMD. The parameter number of the DNN and learning rate of the optimizer as hyperparameters were manually and appropriately tuned during multiple rounds of training and validation to control the behavior of the neural networks. Stochastic gradient descent was used as the optimization algorithm [34]. A fully connected layer was used as the output layer. The DNN was built by training the DXA‐derived BMD values as the ground truth of the training data and preprocessed X‐ray images.

### 2.6 Evaluating the Performances of the ANN

The lumbar and femoral data sets were randomly divided into five groups to avoid including the same participants in the training and test data sets. Five‐fold cross‐validation was performed to evaluate the accuracy of the estimated BMD using the ANN. The four groups of training data sets of the X‐ray images were correlated with the DXA‐derived BMD values, which were input into each DNN for the lumbar and femur, and the trained parameters were constructed in each DNN. After training, only the X‐ray images in the untrained data set group were input to each trained DNN as a test data set for estimation, and the estimated lumbar and femoral BMD values were the output. The training and testing processes were repeated five times for each group of data sets. The DXA‐derived and AI‐estimated T‐scores of the lumbar and femur, based on the Japanese osteoporosis diagnostic criteria [8], were calculated from the DXA‐derived and AI‐estimated BMD values, respectively. The participants were classified into three categories according to criteria by body mass index (BMI) (kg/m^2^) [35]: underweight (BMI < 18.5), normal (18.5 ≤ BMI < 25.0), and overweight (25.0 ≤ BMI). To evaluate the attention area of the ANN, color images were outputted as attention heat maps from the information of the attention layer in the transformer‐based network. All experiments and evaluations were performed using PyTorch 1.13.0 (https://pytorch.org/) as a framework for constructing neural networks on a Windows 10 machine (Microsoft Corporation, Redmond, WA) with a Central Processing Unit and Graphics Processing Unit (TITAN RTX, NVIDIA Corporation, Santa Clara, CA).

Furthermore, the association between the degree of spinal deformity and sclerosis and the estimation accuracy was examined. The degree of spinal deformity and sclerosis was assessed by a single experienced orthopedist (S.M.), who was blinded to each patient's background, using the Kellgren–Lawrence (KL) grading at each intervertebral level from L1/2 to L5/S [36, 37, 38]. The KL scale defines radiographic osteoarthritis in five categories (KL 0–4). The maximum KL grade across the five intervertebral levels (L1/2 to L5/S) was used to group participants into the categories.

### 2.7 Statistical Analysis

The estimation performance of the ANN for lumbar and femoral BMD was evaluated by calculating the mean absolute error (MAE) and the correlation coefficient (*r*) between BMD values derived from DXA and those estimated by AI. Additionally, T‐scores for the lumbar spine and femur were calculated from the estimated BMD values, and participants were classified into normal, osteopenia, and osteoporosis groups. To assess classification performance, sensitivity, specificity, accuracy, positive predictive value (PPV), and area under the receiver operating characteristic curve (AUC) were calculated. Furthermore, to examine the relationship between the KL grade and BMD estimation accuracy, the Jonckheere–Terpstra test was applied using the maximum KL grade (KL 0–4) as an ordinal grouping variable. This test, like the Kruskal–Wallis test, is a nonparametric method, but it has greater statistical power when a monotonic trend (increasing or decreasing) is hypothesized across ordered groups [39, 40]. All analyses were performed using Python 3.8.18 (https://www.python.org) and JMP Pro 18 (SAS Institute Inc.).

## 3 Results

The total number of radiographs classified by sex, BMI category (underweight, normal, and overweight), and DXA‐derived BMD category (normal, osteopenia, and osteoporosis) are shown in Table 1. A total of 1454 X‐ray images from 1454 participants were analyzed for the lumbar and femoral BMD estimations using each DNN.

**Table 1:** Characteristics of the lumbar and femoral data set overall by sex, BMI, disease category, and imaging device.

		Total	Sex		BMI			Manufacturer	
			Female	Male	Underweight	Normal	Overweight	FUJIFILM	KONICA MINOLTA
Number of X‐ray images		1454	964	490	112	963	379	211	1,243
Age mean (years)		65.6	65.3	66.1	65.3	65.8	65.0	71.6	64.5
(Min–Max)		(19–94)	(19–94)	(20–93)	(19–88)	(25–94)	(20–92)	(39–94)	(19–93)
BMI mean (kg/m^2^)		23.0	22.7	23.6	17.5	21.9	27.7	22.8	23.1
(Min–Max)		(13.1–46.6)	(13.1–43.7)	(15.9–46.6)	(13.1–18.5)	(18.5–25.0)	(25.0–46.6)	(14.6–35.1)	(13.1–46.6)
Lumbar data set									
BMD mean (g/cm^2^)		0.958	0.893	1.087	0.821	0.942	1.040	0.933	0.963
(Min–Max)		(0.428–1.868)	(0.428–1.657)	(0.591–1.868)	(0.441–1.292)	(0.428–1.666)	(0.542–1.868)	(0.428–1.545)	(0.441–1.868)
T‐score mean		−0.4	−1.0	0.6	−1.6	−0.6	0.2	−0.7	−0.4
(Min–Max)		(−4.9 to 7.2)	(−4.9 to 5.4)	(−3.5 to 7.2)	(−4.8 to 2.4)	(−4.9 to 5.5)	(−3.9 to 7.2)	(−4.9 to 4.5)	(−4.8 to 7.2)
Categories, *n* (%)	Normal	875 (60.2)	459 (47.6)	416 (84.9)	42 (37.5)	550 (57.1)	283 (74.7)	114 (54.0)	761 (61.2)
	Osteopenia	434 (29.8)	367 (38.1)	67 (13.7)	33 (29.5)	322 (33.4)	79 (20.8)	73 (34.6)	361 (29.0)
	Osteoporosis	145 (10.0)	138 (14.3)	7 (1.4)	37 (33.0)	91 (9.4)	17 (4.5)	24 (11.4)	121 (9.7)
Femoral data set									
BMD mean (g/cm^2^)		0.664	0.621	0.748	0.569	0.650	0.727	0.631	0.670
(Min–Max)		(0.267–1.273)	(0.267–1.086)	(0.392–1.273)	(0.267–0.926)	(0.328–1.216)	(0.347–1.273)	(0.345–1.044)	(0.267–1.273)
T‐score mean		−1.4	−1.9	−0.5	−2.5	−1.6	−0.7	−1.8	−1.3
(Min–Max)		(−5.8 to 5.4)	(−5.8 to 3.3)	(−4.4 to 5.4)	(−5.8 to 1.5)	(−5.1 to 4.7)	(−4.9 to 5.4)	(−4.9 to 2.8)	(−5.8 to 5.4)
Categories, *n* (%)	Normal	518 (35.6)	211 (21.9)	307 (62.7)	17 (15.2)	300 (31.2)	201 (53.0)	55 (26.1)	463 (37.2)
	Osteopenia	605 (41.6)	460 (47.7)	145 (29.6)	39 (34.8)	428 (44.4)	138 (36.4)	89 (42.2)	516 (41.5)
	Osteoporosis	331 (22.8)	293 (30.4)	38 (7.8)	56 (50.0)	235 (24.4)	40 (10.6)	67 (31.8)	264 (21.2)

Abbreviations: BMD, bone mineral density; BMI, body mass index.

**Figure 3:**
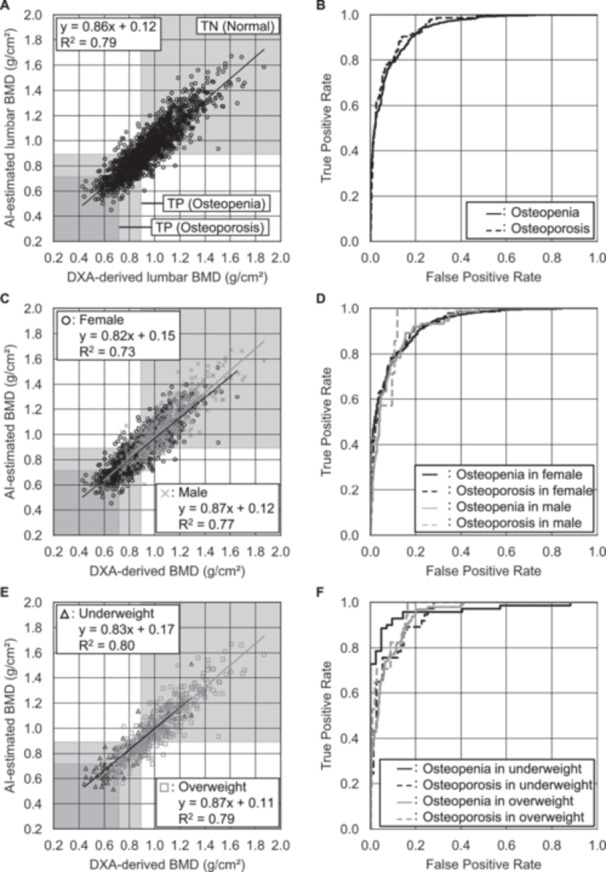
Lumbar BMD estimation and osteoporosis classification performances in all estimations by sex and by BMI categories. Correlation scatter plots (A, C, E) and ROC curves (B, D, F) for estimated BMDs of all patients, female, and male, respectively. AI, artificial intelligence; AUCs, areas under the curve; BMD, bone mineral density; PPVs, positive predictive values; ROC, receiver operating characteristic; TN, true negative; TP, true positive.

**Figure 4:**
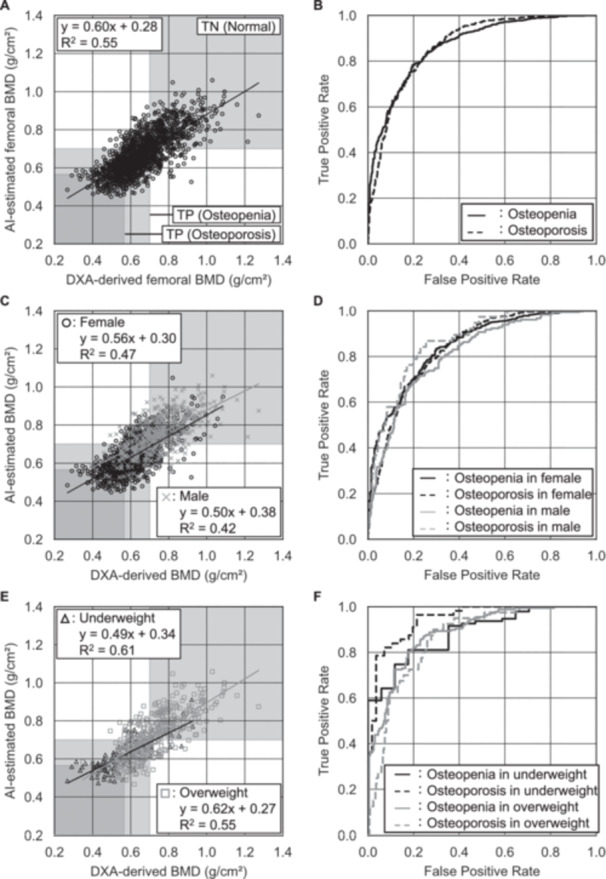
Femoral BMD estimation and osteoporosis classification performances in all estimations by sex and by BMI categories. Correlation scatter plots (A, C, E) and ROC curves (B, D, F) for estimated BMDs for the underweight, normal, and overweight patients, respectively. (ROC: receiver operating characteristic. AI, artificial intelligence; AUCs, areas under the curve; BMD, bone mineral density; BMI, body mass index; PPVs, positive predictive values; ROC, receiver operating characteristic; TN, true negative; TP, true positive.

Regarding the BMD estimation performance using the ANN, the MAEs, mean relative errors (MREs), and correlation coefficients in the BMD estimations of all participants were 0.076 g/cm^2^, 8.1%, and 0.89 between DXA‐derived and AI‐estimated lumbar BMD values, and 0.071 g/cm^2^, 11.3%, and 0.74 for femoral BMD values, respectively (Table 2). The DXA‐derived and AI‐estimated lumbar BMD values were strongly correlated (Figure 3A) regardless of sex and BMI (Figure 3C,E). A correlation between DXA‐derived and AI‐estimated values was also observed in femoral BMD estimations (Figure 4A), regardless of sex and BMI (Figure 4C,E). The MAEs for lumbar and femoral BMD estimations in male participants (0.081 and 0.079 g/cm^2^, respectively) were larger than those in female participants (0.073 and 0.067 g/cm^2^, respectively). Conversely, the MREs in male participants (7.5% and 10.9%) were almost the same as or smaller than those in female participants (8.4% and 11.6%, respectively). Each MAE was comparable between the lumbar and femoral BMD estimations across all BMI categories. The MREs demonstrated a decreasing trend with increasing BMI. Conversely, the MREs increased with the severity of osteoporosis. MREs in the femoral BMD estimations were consistently higher than those in the lumbar estimations, regardless of sex or osteoporosis category. Conversely, the correlation coefficients in the femoral BMD estimations were consistently lower than those in the lumbar estimations across all subgroups, including sex and osteoporosis categories.

**Table 2:** Lumbar and femoral BMD estimation and osteoporosis classification accuracy overall using an ANN by sex, BMI, disease category, and imaging device.

		Total	Sex		BMI			Manufacturer		Disease categories		
			Female	Male	Underweight	Normal	Overweight	FUJIFILM	KONICA MINOLTA	Normal	Osteo‐penia	Osteo‐porosis
Lumbar												
MAE (g/cm^2^)		0.076	0.073	0.081	0.069	0.075	0.079	0.080	0.075	0.081	0.065	0.073
MRE (%)		8.1	8.4	7.5	9.5	8.1	7.7	9.0	7.9	7.4	8.1	11.9
Correlation coefficient		0.89	0.85	0.88	0.89	0.88	0.89	0.87	0.89	0.82	0.43	0.36
Osteo‐penia	Sensitivity (%)	86.4	87.5	78.4	91.4	86.2	83.3	84.5	86.7	NA	82.3	98.6
	Specificity (%)	84.1	80.4	88.2	90.5	82.5	86.2	82.5	84.4	84.1	NA	NA
	Accuracy (%)	85.0	84.1	86.7	91.1	84.1	85.5	83.4	85.3	84.1	82.3	98.6
	PPV (%)	78.2	83.1	54.2	94.1	78.8	67.2	80.4	77.8	0	100.0	100.0
	AUC	0.937	0.929	0.925	0.960	0.931	0.942	0.940	0.936	NA	NA	NA
Osteo‐porosis	Sensitivity (%)	71.7	73.2	42.9	70.3	73.6	64.7	75.0	71.1	NA	NA	71.7
	Specificity (%)	94.7	92.5	98.6	94.7	93.3	98.1	92.0	95.2	99.9	84.3	NA
	Accuracy (%)	92.4	89.7	97.8	86.6	91.5	96.6	90.0	92.8	99.9	84.3	71.7
	PPV (%)	60.1	62.0	30.0	86.7	53.6	61.1	54.5	61.4	0	0	100.0
	AUC	0.945	0.931	0.949	0.940	0.942	0.955	0.921	0.949	NA	NA	NA
Femur												
MAE (g/cm^2^)		0.071	0.067	0.079	0.079	0.070	0.074	0.076	0.071	0.077	0.060	0.084
MRE (%)		11.3	11.6	10.9	16.4	11.1	10.4	12.8	11.1	9.3	9.6	17.8
Correlation coefficient		0.74	0.69	0.65	0.78	0.70	0.74	0.70	0.75	0.50	0.35	0.30
Osteo‐penia	Sensitivity (%)	80.4	85.1	61.2	90.5	80.7	74.2	85.3	79.5	NA	71.4	97.0
	Specificity (%)	76.3	65.4	83.7	64.7	72.0	83.6	74.5	76.5	76.3	NA	NA
	Accuracy (%)	79.0	80.8	75.3	86.6	78.0	79.2	82.5	78.4	76.3	71.4	97.0
	PPV (%)	86.0	89.8	69.1	93.5	86.4	80.0	90.5	85.0	0	100.0	100.0
	AUC	0.868	0.846	0.813	0.885	0.846	0.884	0.877	0.865	NA	NA	NA
Osteo‐porosis	Sensitivity (%)	44.4	48.1	15.8	50.0	46.0	27.5	37.3	46.2	NA	NA	44.4
	Specificity (%)	93.8	90.6	98.5	98.2	92.9	95.0	95.1	93.6	99.2	89.1	NA
	Accuracy (%)	82.5	77.7	92.0	74.1	81.4	87.9	76.8	83.5	99.2	89.1	44.4
	PPV (%)	67.7	69.1	46.2	96.6	67.5	39.3	78.1	65.9	0	0	100.0
	AUC	0.869	0.837	0.868	0.945	0.851	0.860	0.815	0.880	NA	NA	NA

Abbreviations: ANN, artificial neural network; AUC, area under the curve; MAE, mean absolute error; MRE, mean relative error; PPV, positive predictive value.

**Figure 5:**
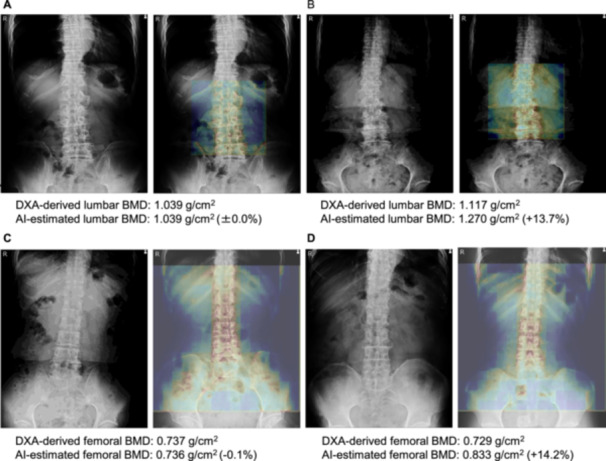
Typical attention heat maps with (A, C) high and (B, D) poor estimation results using an ANN. AI, artificial intelligence; ANN, artificial neural network; BMD, bone mineral density; DXA, dual‐energy X‐ray absorptiometry.

Figure 5 shows the typical attention heat maps overlaid on lumbar X‐ray images with high and poor BMD estimation accuracies using each DNN. The warm‐colored areas focused on by the ANN were primarily used for BMD estimation. In the lumbar BMD estimations with high and poor accuracy (Figure 5A,B, MRE: ±0.0% and +13.7%, respectively), the L1–L4 regions were highlighted with warm colors. In the poor accuracy case (Figure 5B), a compression fracture was noted in L1, along with extensive bone sclerosis and osteophyte formation from L1 to L4. For the femoral BMD estimations, the high accuracy case (Figure 5C, MRE: −0.1%) showed warm color distribution across the thoracic spine, lumbar spine, and pelvis. In contrast, the poor accuracy case (Figure 5D, MRE: +14.2%) exhibited warm color distribution in the thoracic spine, lumbar spine, and ribs.

**Figure 6:**
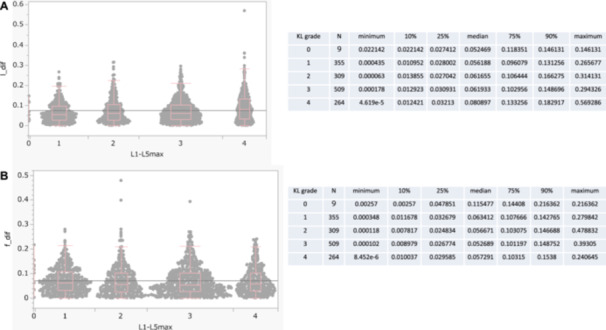
Distribution of absolute differences between AI‐predicted and measured BMD values by KL grade for the lumbar spine and femur. (A) Distribution of absolute differences between AI‐predicted and measured lumbar BMD (l_dif) stratified by KL grade. (B) Distribution of absolute differences between AI‐predicted and measured femoral BMD (f_dif) stratified by KL grade. *l_dif* and *f_dif* represent the differences between the actual DXA measurements and the AI‐predicted values for the lumbar spine and femur, respectively. KL grade was evaluated at each lumbar intervertebral level (L1/2 to L5/S1), and the highest grade per participant was used for analysis. There was a significant monotonic increasing trend in the estimation error of lumbar spine BMD across the KL grade groups (*J* = 418,827, *Z* = 3.819, *p* = 0.0001). This finding indicates that estimation errors tended to increase as the radiographic severity of lumbar spondylosis, as assessed by the KL grade, became more advanced. However, no significant monotonic trend was observed in femoral BMD estimation among the KL grade groups (*J* = 376,019, *Z* = –1.034, *p* = 0.3012), suggesting that the severity of lumbar spondylosis had minimal influence on the accuracy of femoral BMD estimation. AI, artificial intelligence; BMD, bone mineral density; DXA, dual‐energy X‐ray absorptiometry; KL, Kellgren–Lawrence.

The association between the degree of spinal deformity and sclerosis and the estimation accuracy is shown in Figure 6.

## 4 Discussion

This study demonstrated lumbar and femoral BMD estimation and osteoporosis classification from a plain lumbar anteroposterior X‐ray image using deep learning. This study's findings provide preliminary evidence that our AI‐assisted osteoporosis diagnosis system estimates lumbar and femoral BMD and distinguishes patients with osteopenia and osteoporosis from ordinary inhabitants.

We can assume that whether the input X‐ray image includes an estimated object or not is an important factor for BMD estimation and classification performance. The femoral BMD estimation and classification performance did not perform as well as those for the lumbar region. This is attributed to the femur not being included in the lumbar X‐ray image, and the ANN could not directly estimate the femoral BMD. However, even the femoral BMD estimation and classification performance of this study was equivalent to the conventional BMD measurement equipment reported in previous studies: the correlation coefficient, sensitivity, and specificity of radial DXA were approximately 0.46–0.69 [41, 42], 79%, and 71% [43]; those of radiographic absorptiometry were approximately 0.66%, 83%, and 68% [44]; and those of QUS were approximately 0.14%–0.60%, 49%–86%, and 36%–83% [44, 45, 46, 47, 48, 49], respectively. Similar sensitivities and specificities to conventional diagnostic systems suggest that this AI‐assisted diagnosis system could become an effective osteoporosis screening tool for the lumbar spine and femur.

The MAEs in male participants were larger than those in female participants for lumbar and femoral BMD estimations. This may be attributed to the higher BMD values in men than in women, which can lead to greater absolute errors (MAEs) even when the prediction accuracy is comparable. However, the MREs in men were approximately equal to or lower than those in women. A similar trend was observed in overweight participants and those with normal BMD. Conversely, the opposite tendency was observed in underweight participants and those with osteoporosis, indicating that the prediction errors tended to be larger in low BMD groups. These differences are not attributable to the performance of the ANN, but rather to group bias. These findings suggest that the proposed system may be useful for most individuals, regardless of sex, BMI, or osteoporosis categories.

The FUJIFILM and KONICA devices demonstrated favorable estimation accuracy across key metrics. Notably, the AUCs for all classification tasks exceeded 0.8, indicating that while further improvement in precision is expected in future studies, the current method has already achieved a clinically applicable level of performance. Although minor differences in performance indicators were observed between the two devices, these are likely attributable to differences in participant characteristics—particularly age distribution and baseline bone density levels. The KONICA group had a higher mean age and a greater proportion of participants with osteoporosis than the FUJIFILM group, which may have contributed to slightly lower sensitivity and higher estimation difficulty in the KONICA group. Nevertheless, the BMD estimation results were consistent across both devices, and the device‐related differences in performance were not considered to have clinically significant impact. In particular, lumbar BMD estimations showed high correlation coefficients and robust AUC values, suggesting that both systems provide stable and reliable predictions. Taken together, the findings suggest that accurate and reliable BMD estimation can be achieved regardless of which imaging device is used.

Regarding classification performance, the sensitivities and PPVs for participants with osteoporosis were lower than those for other osteoporosis categories in lumbar and femoral BMD estimations. This tendency was particularly pronounced among male participants. Although the model did not incorporate sex as an input, performance evaluation by sex remained meaningful, as anatomical differences may be implicitly reflected in the image data. This system estimated BMD using only image data, without directly considering sex in its analysis or diagnosis. This design was chosen to promote generalizability and avoid reliance on demographic information that may not always be available. However, differences in model performance between male and female participants can still occur due to indirect cues present in image data and imbalances in training data distribution. This issue arises from the fact that the present study was a cohort study targeting residents of a specific region, leading to an imbalance in participant distribution. The prevalence of osteoporosis is substantially lower in men than in women [50]. When using data from clinical settings, osteoporosis testing is performed on individuals suspected of having the disease; thus, a certain proportion of male patients with osteoporosis is expected. However, in this study, BMD testing was conducted in all residents, including healthy individuals. Consequently, the number of male participants with osteoporosis was limited. In contrast, the number of female participants with osteoporosis was notably higher, further highlighting the class imbalance. Imbalanced data sets tend to bias classifiers toward the majority class, leading to reduced performance in detecting important minority cases [51]. This issue arises because conventional machine learning algorithms are optimized to minimize overall training error, often at the expense of minority class accuracy. In our study, the ANN was trained to accurately estimate BMD values across most of the data set, which predominantly included individuals with average BMD. Consequently, classification performance for the minority class—participants with osteoporosis—was degraded in lumbar and femoral cases. Nevertheless, the MAEs for lumbar and femoral BMD estimations in participants with osteoporosis were nearly equivalent to those observed in healthy participants and those with osteopenia. This suggests that while the classification thresholds were less effective for the minority class, the regression‐based estimation of BMD itself remained consistent across groups. To address these limitations, we plan to enhance data balance through oversampling or data augmentation, and to explore cost‐sensitive learning strategies that prioritize minority class accuracy. Although we do not plan to include sex explicitly as an input feature, we acknowledge the importance of subgroup analyses in evaluating fairness and robustness of the model. Such an approach is expected to enhance classification performance without compromising the regression accuracy already achieved.

Several cases of poor estimation accuracy were observed, even when the ANN focused on the L1–L4 vertebral region (Figure 5B). This may be due to the presence of a compression fracture in L1, along with extensive bone sclerosis and osteophyte formation from L1–L4. The ANN shown in Figure 5D gave less attention to the pelvic area than the ANN shown in Figure 5C. This limited focus may have contributed to the inaccurate estimation of femoral BMD (Figure 5D). The pelvis is considered more critical than the ribs in estimating femoral BMD, as its morphology reflects important factors that affect BMD values [52]. This emphasizes the importance of pelvic features in femoral BMD estimation.

A significant monotonic increase in estimation error was observed in lumbar spine BMD as the severity of lumbar spondylosis increased, whereas no such trend was found in femoral BMD estimation. This suggests that spinal degenerative changes affect the accuracy of lumbar, but not femoral, BMD estimation. In the lumbar spine, osteophyte formation and vertebral sclerosis may obscure trabecular bone structure on radiographs, introducing artifacts that interfere with the features used by the AI system. These findings are consistent with those of previous reports, which have shown that spinal degeneration can compromise the accuracy of DXA‐based BMD measurements [53]. In contrast, during the early stages of AI development, it was initially assumed that such degenerative changes might also affect femoral BMD estimation, resulting in overestimation relative to DXA. However, with continued model improvements and as demonstrated in the current study, spinal degeneration appears to have minimal influence on femoral BMD estimation. This indicates that AI models focused on the femur can maintain reliable performance regardless of spinal degenerative status.

Although these findings are promising, some methodological limitations should be considered. First, although we evaluated a data set from a population‐based cohort study, the clinical imaging conditions were limited. The X‐ray images were obtained using only two types of imaging equipment and two imaging protocols. While the present evaluation included data sets from various imaging devices and procedures, the generalizability of the results may still be limited. Additionally, data augmentation techniques were used to simulate radiographic variation, but further studies are needed to determine whether differences in imaging conditions across institutions affect BMD estimation and osteoporosis classification. Second, only anteroposterior lumbar radiographs were used, and basic demographic information was not incorporated into the model. Although these factors influenced bone density, we did not evaluate their impact. Nonetheless, achieving high estimation accuracy from a single image remains a notable strength of our approach. Third, there was a partial mismatch between the KL evaluation and the AI estimation regions. KL grading was conducted at five intervertebral levels (L1/2 to L5/S1), while AI‐based lumbar BMD estimation used the L1–L4 region. Moreover, we used the maximum KL grade among the five levels to assess degenerative severity. While this method captures the most advanced degenerative change, it may not fully reflect the overall burden. Using a cumulative metric could provide a more comprehensive assessment. Fourth, although vertebral compression fractures were identified during image review, neither their presence nor number was included in the analysis. As compression fractures can alter vertebral morphology and potentially influence AI‐based prediction, their exclusion may limit the interpretability and clinical applicability of the model. Fifth, the number of cases from the FUJIFILM group were substantially larger than those from the KONICA group, accounting for approximately 85% of the total data set. This imbalance in sample size may have influenced the training and evaluation of the model, potentially introducing bias toward the data characteristics of the FUJIFILM group. Performance indicators obtained from the KONICA group may be subject to greater variability and should be interpreted with caution. Sixth, the imaging data were collected from specific facilities under standardized conditions. Therefore, further validation using external data sets from diverse institutions is needed to ensure the broader applicability and robustness of the proposed method.

## 5 Conclusions

We demonstrated that this AI‐assisted osteoporosis diagnostic system, using an anteroposterior lumbar X‐ray image, could estimate the BMD of the lumbar spine and femur and classify patients with osteopenia and osteoporosis. The estimation accuracy for femoral BMD was acceptable, although lower than that for lumbar BMD. This AI system was able to estimate BMD and classify osteoporosis categories not only in patients from clinical or hospital settings but also in the general population, including healthy individuals. Our AI‐assisted osteoporosis diagnostic system using lumbar X‐ray images is expected to increase opportunities to (a) identify potential patients with osteopenia and osteoporosis, (b) facilitate early detection of high‐risk individuals, and (c) help prevent bone loss and fragility fractures in areas, such as the lumbar spine and femur, by encouraging improvements in lifestyle habits, including diet and exercise.

## Author Contributions

**Toru Moro:** conceptualization, project administration, writing – original draft. **Noriko Yoshimura:** data curation, resources. **Taku Saito:** validation, supervision. **Hiroyuki Oka:** statical analysis. **Shigeyuki Muraki:** data curation. **Toshiko Iidaka:** data curation, resources. **Takeyuki Tanaka:** data curation, validation. **Kumiko Ono:** data curation, validation. **Hisatoshi Ishikura:** data curation, validation. **Naoya Wada:** software, formal analysis, methodology, investigation. **Kenichi Watanabe:** formal analysis, methodology, validation. **Masayuki Kyomoto:** conceptualization, project administration, writing – review and editing. **Sakae Tanaka:** supervision, funding acquisition. All authors have read and approved the final submitted manuscript.

## Conflicts of Interest

One or more of the authors or institutions received outside funding or grants from the KYOCERA Corporation. One or more of the authors (N.W., K.W., and M.K.) are employed by the KYOCERA Corporation.
